# Book Review: Handbook of Positive Youth Development: Advancing the Next Generation of Research, Policy, and Practice in Global Contexts

**DOI:** 10.3389/fpsyg.2021.716388

**Published:** 2021-08-18

**Authors:** Arzu Karakulak, Sena Cüre-Acer

**Affiliations:** Department of Psychology, Bahcesehir University, Istanbul, Turkey

**Keywords:** PYD, 5Cs 6Cs and 7Cs model, developmental assets, adolescents, emerging adults, research, policy, practice

Adolescents and emerging adults are the two populations most affected by the changing world (Dimitrova, 2018; Mehta et al., 2020), and their well-being is critical for the social well-being globally. Even though Positive Youth Development PYD scholars have long acknowledged that different contexts and conditions in youths' life provide different opportunities for learning (Burkhard et al., 2019; Shek et al., 2019; Syvertsen et al., 2019; Wiium and Dimitrova, 2019) research about the adaptability, utility, and limitations of various PYD conceptualizations and practices across diverse (cultural) contexts remained relatively understudied. The Handbook of Positive Youth Development: Advancing the Next Generation of Research, Policy and Practice in Global Contexts edited by Dimitrova and Wiium (2021) tackles this need and offers an integrative and comprehensive collection of empirical evidence about the conceptualization and application of PYD approaches across the globe. A relevant feature of this volume is to expand the generalizability of the PYD framework beyond the WEIRD contexts by presenting a set of empirical findings from innovative theoretical and methodological approaches (e.g., cross-cultural, multi-national, experimental, longitudinal mixed-methods) enlarging the broad literature with fresh international perspectives. In so doing, each chapter of the volume presents a context specific description of the local socio-political, cultural features and current issues affecting youth, PYD programs, and studies for policy and intervention. Most importantly, context/cultural specifics and universal mechanisms of PYD are presented delineating what is unique in each chapter and how the knowledge provided can also be useful to understand and apply to other contexts, while balancing between non-WEIRD (i.e., Albania, Bulgaria, Czech Republic, Kosovo, Serbia, Romania, Ghana, South Africa) and WEIRD (USA, Canada, Italy) contexts.

The *handbook* is unique and unprecedented in its' scope as (1) it covers evidence based on various conceptualizations and indices of PYD that outline the holistic and interactive nature of PYD; (2) presents PYD evidence far beyond the WEIRD contexts drawing attention to extraordinary samples and their specific needs; (3) addresses issues of cross-cultural measurement invariance, and (4) bridges research and practice on PYD across the globe that enables the dissemination of knowledge for improving the lives of youth.

## Holistic PYD Conceptualizations

The models proposed by PYD research touch many different areas of youths' and emerging adults' lives. Likewise, the current *handbook* understands PYD as an overarching and dynamic process, and presents evidence on how key concepts such as identity, school and student engagement, mindfulness, self-efficacy, and hopeful future expectations interact with contextual resources such as parents, peers, and schools to provide the bases of PYD. The chapters of the current *handbook* situate and study these processes within two prominent PYD frameworks: the 5Cs and 6Cs model (Geldhof et al., 2015; Burkhard et al., 2019), and the developmental assets model (Scales et al., 2017). With the evidence presented in the *handbook*, it is now visible that these models touch on globally valid mechanisms of PYD. Most innovatively, the *handbook* expands existing models, and presents evidence on the cross-cultural utility of the 7Cs model of PYD by proposing a new indicator, *creativity*; an important component that shows youths' strength on their ability to adapt to novel situations (Abdul Kadir et al., 2021; Dimitrova and Wiium, 2021; Dimitrova et al., 2021a; Manrique-Millones et al., 2021).

The *handbook* presents evidence on how various conceptualizations of PYD associate with healthy lifestyle behaviors (Dominguez et al., 2021), and improve mindfulness among emerging adults (Abdul Kadir et al., 2021). It demonstrates the relationship between the 5Cs of PYD and environmental concerns (Kabir and Wiium, 2021), happiness (Gomez-Baya et al., 2021), hopeful expectations about future and life satisfaction (Fernandes et al., 2021), and outlines how both internal and external assets contribute to PYD (Dost-Gözkan and Wiium, 2021). Various chapters draw on the interplay between internal and external assets for PYD. They illustrate the relationship between internal assets of PYD and social support (Kosic et al., 2021), outline the role of parents (e.g., Dutra-Thomé and Ponciano, 2021) and the school environment (Ginner Hau et al., 2021) as an external asset for healthy youth development, but also underline the importance of internal assets such as self-esteem to promote life satisfaction among youth, especially when faced with adverse experiences such as domestic violence or abuse (Ásgeirsdóttir and Sigfúsdóttir, 2021). The *handbook* thus illustrates relevant person-context interactions in the domain of PYD and demonstrates that PYD represents a multifaceted process that cannot be reduced to individual-level strengths, but must be understood dynamically in relation to external resources such as schools (Acosta et al., 2021; Bradley et al., 2021; Ginner Hau et al., 2021), parents (Dutra-Thomé and Ponciano, 2021; Lansford et al., 2021; McKee et al., 2021), youth programs and interventions (Kaniušonyte and Truskauskaite-Kunevičiene, 2021; Kozina, 2021; Larsen and Holsen, 2021; Wang et al., 2021), and socio-cultural boundary conditions (James et al., 2021; Smith et al., 2021; Uka et al., 2021).

## Cross-Cultural Approach

The *handbook* comprises data from a wide array of samples with 37 empirical chapters with data from across 38 countries with a total number of 22,083 responses. A particular focus lies on understanding the process of PYD in understudied contexts to offer a global view on PYD that both comprises and expands beyond the widely studied Western contexts. Among others, the *handbook* presents evidence from India, Indonesia and Pakistan (Dimitrova et al., 2021a), Malaysia (Abdul Kadir et al., 2021), Columbia and Peru (Manrique-Millones et al., 2021), Brazil (Dutra-Thomé and Ponciano, 2021), Ghana (Wiium and Kozina, 2021), Mexico (Dominguez et al., 2021), Turkey (Dost-Gözkan and Wiium, 2021), Lithuania (Kaniušonyte and Truskauskaite-Kunevičiene, 2021), Romania (Dimitrova et al., 2021b), and South Africa (Bremner and Schwartz, 2021). A core attention is also given to understanding the process of PYD among underrepresented minority groups, as illustrated for instance in the chapter of Kosic et al. (2021) who examined the process of PYD among Slovene minority youth living in Italy and Uka et al. (2021) who studied PYD processes among Albanians living in Albania, Kosovo, Macedonia and Serbia.

The data presented in the current volume do not only vary nationally and ethnically, but also socially. The chapter by Negru-Subtirica and Badescu (2021) outlines social factors may affect PYD by analyzing how macrosocial and political indicators, as well as intergenerational transmission through families shape youth vocational competence. The chapter by Hull et al. (2021) further outlines how social factors may affect the process of PYD. Hull et al. (2021) conducted a longitudinal field experiment with NEET (Not active in Education, Employment, or Training opportunities) Jamaican youth, and found that the National Youth Service Corps program promoted their career decision self-efficacy over time; however, the effects of the program for less skilled youth representing the lower stratum of NEET remained rather limited. Likewise, Eichas et al. (2021) draw attention to the role of social factors by presenting narrative case histories from the Changing Lives Program designed to empower marginalized adolescents growing up in less advantaged community contexts in Miami, Florida, the USA.

Despite diversity of operationalization and applications, the *handbook* illustrates that PYD models for understanding and strengthening positive youth development-the developmental assets (Scales et al., 2017) and the 5Cs and 6Cs models of PYD (Geldhof et al., 2015) and the newly developed 7C model (Dimitrova et al., 2021a)- share similarities in the emphases on youth strengths and potentials as well as internal and external developmental assets. By applying a PYD approach, this volume documents what social, cultural and educational challenges youth encounter in a number of societies across the globe. This *volume* incorporates relevant efforts outlining both universalities and specifics of development by providing theoretical perspectives with supporting empirical findings to promote a better understanding and insights into youth thriving. The book therefore provides the reader with a better understanding of the conditions which foster optimal development of youth embedded within mediating/moderating contributions of family, community, social and cultural factors. Specifically for youthful populations, the book indicates the pathways in which youth cope with their cultural, social and educational challenges and cultural and contextual factors that foster their well-being.

## Methodological Advancements

Not only conceptually but also methodologically, the *handbook* underscores that PYD represents a common matter across diverse cultural, ethnic, and socio-economical contexts, and yet may demand context-specific adaptations (Dimitrova et al., 2021c). The research reported in the *handbook* and the analyses conducted adhere to the highest standards of scientific excellence with particular focus on cultural adaptation and measurement invariance issues (Dimitrova and Wiium, 2021; Dimitrova et al., 2021b). Invariance tests are concerned with the question of comparability across cultures, and a provide a guide as to what extent (a) the content and general structure of measurement concepts, (b) its measurement procedures and items, and (c) the scores achieved on a measure are cross-culturally invariant (and thus comparable) or demand culture-specific adaptations (van de Vijver, 2019). As such, the current *handbook* helps to identify and develop psychometrically strong and cross-culturally applicable measures of PYD and therefore will facilitate and promote further global research on PYD.

## Bridging Research, Policy and Practice

A major milestone of the volume is to bridge both basic research in part I, and more applied research examining the impact of interventions in part II. The related research findings and empirical illustrations are communicated in a way that enables multidisciplinary inexpert audience to understand the findings, thus easily facilitating fruition by lay people, policy makers and practitioners. In so doing, the volume emphasizes the multidisciplinary approach to PYD, since it is important that professionals from a variety of disciplines (e.g., cross-cultural and developmental psychology, applied developmental science) work together in PYD practice and research, for the benefit of youth and emerging adults. With all that extent, the *handbook* not only provides academic knowledge, but it also informs about how the knowledge on PYD can be applied in the real-world for youth and emerging adults. A core foundation of this *handbook* is to overcome all issues related to inequity, stereotypes, and social justice toward minorities. There are relevant chapters addressing a variety of ethnic minority groups subjected to marginalization and discrimination. The book stands apart from current edited books on PYD by focusing on these ethnic minority youth and on strengths and resources for optimal well-being. In such ways, the book presents a knowledge-base for research and practice with minority youth based on a PYD strength-based conception of development in contrast to traditional deficit models seeing these youth as problematic and dysfunctional.

## Conclusion

The reviewed *handbook* advances nuanced understanding of PYD among culturally diverse young populations by providing a global perspective. In so doing, the volume focuses on the strengths, potentials and contributions of youth and emerging adults, and generates a positive view to the field and highlights the psychological and social factors as well their dynamics that shape PYD, and eventually contribute toward achieving a more positive future across various societies (Geldhof et al., 2021). Importantly, this book is the first of its kind to present the most comprehensive collection of PYD contributions to broader contexts and global audiences beyond the WEIRD vs. non-WEIRD dichotomy. This volume views this as an imperative, given that, with a few notable exceptions, a majority of the current knowledge base derives from White, Western, educated, industrialized, democratic and rich (WEIRD) countries neglecting relevant settings around the globe.

The *handbook* combines evidence about the adaptability, utility, and limitations of various PYD conceptualizations and practices, and becomes an important guideline in shaping PYD's future in research, policy and practice across the globe. It does so conceptually by assembling the voices of young scholars who conduct PYD research in underrepresented cultural contexts with understudied samples, and it does so methodologically by raising the issue of measurement invariance to decide whether and to what extent existing measures can be adapted to new contexts (Lansford et al., 2021). As such, the current *handbook* is a prime example of how to navigate PYD research and practices toward considering the specifics of PYD while not losing the view for its' universals.

## Author Contributions

All authors listed have made a substantial, direct and intellectual contribution to the work, and approved it for publication.

## Conflict of Interest

The authors declare that the research was conducted in the absence of any commercial or financial relationships that could be construed as a potential conflict of interest.

## Publisher's Note

All claims expressed in this article are solely those of the authors and do not necessarily represent those of their affiliated organizations, or those of the publisher, the editors and the reviewers. Any product that may be evaluated in this article, or claim that may be made by its manufacturer, is not guaranteed or endorsed by the publisher.
